# Involvement of PPAR-γ in the neuroprotective and anti-inflammatory effects of angiotensin type 1 receptor inhibition: effects of the receptor antagonist telmisartan and receptor deletion in a mouse MPTP model of Parkinson's disease

**DOI:** 10.1186/1742-2094-9-38

**Published:** 2012-02-22

**Authors:** Pablo Garrido-Gil, Belen Joglar, Ana I Rodriguez-Perez, Maria J Guerra, Jose L Labandeira-Garcia

**Affiliations:** 1 Laboratory of Neuroanatomy and Experimental Neurology, Department of Morphological Sciences, Faculty of Medicine, University of Santiago de Compostela, Santiago de Compostela, Spain; 2 Networking Research Center on Neurodegenerative Diseases (CIBERNED), Santiago de Compostela, Spain

**Keywords:** Angiotensin, AT1, neuroinflammation, neuroprotection, microglia, Parkinson, peroxisome proliferator-activated receptor gamma, telmisartan

## Abstract

**Background:**

Several recent studies have shown that angiotensin type 1 receptor (AT1) antagonists such as candesartan inhibit the microglial inflammatory response and dopaminergic cell loss in animal models of Parkinson's disease. However, the mechanisms involved in the neuroprotective and anti-inflammatory effects of AT1 blockers in the brain have not been clarified. A number of studies have reported that AT1 blockers activate peroxisome proliferator-activated receptor gamma (PPAR γ). PPAR-γ activation inhibits inflammation, and may be responsible for neuroprotective effects, independently of AT1 blocking actions.

**Methods:**

We have investigated whether oral treatment with telmisartan (the most potent PPAR-γ activator among AT1 blockers) provides neuroprotection against dopaminergic cell death and neuroinflammation, and the possible role of PPAR-γ activation in any such neuroprotection. We used a mouse model of parkinsonism induced by the dopaminergic neurotoxin 1-methyl-4-phenyl-1,2,3,6-tetrahydropyridine (MPTP) and co-administration of the PPAR-γ antagonist GW9662 to study the role of PPAR-γ activation. In addition, we used AT1a-null mice lesioned with MPTP to study whether deletion of AT1 in the absence of any pharmacological effect of AT1 blockers provides neuroprotection, and investigated whether PPAR-γ activation may also be involved in any such effect of AT1 deletion by co-administration of the PPAR-γ antagonist GW9662.

**Results:**

We observed that telmisartan protects mouse dopaminergic neurons and inhibits the microglial response induced by administration of MPTP. The protective effects of telmisartan on dopaminergic cell death and microglial activation were inhibited by co-administration of GW9662. Dopaminergic cell death and microglial activation were significantly lower in AT1a-null mice treated with MPTP than in mice not subjected to AT1a deletion. Interestingly, the protective effects of AT1 deletion were also inhibited by co-administration of GW9662.

**Conclusion:**

The results suggest that telmisartan provides effective neuroprotection against dopaminergic cell death and that the neuroprotective effect is mediated by PPAR-γ activation. However, the results in AT1-deficient mice show that blockage of AT1, unrelated to the pharmacological properties of AT1 blockers, also protects against dopaminergic cell death and neuroinflammation. Furthermore, the results show that PPAR-γ activation is involved in the anti-inflammatory and neuroprotective effects of AT1 deletion.

## Background

In recent years, evidence has accumulated for a major role of oxidative stress and neuroinflammation in the pathogenesis and progression of Parkinson's disease (PD) [1,2]. The peptide angiotensin II (AII), via type 1 receptors (AT1), is one of the most important known inducers of inflammation and oxidative stress, produces reactive oxygen species (ROS) by activation of the reduced nicotinamide adenine dinucleotide phosphate (NADPH)-oxidase complex [3-5] and plays a major role in the pathogenesis of several age-related degenerative diseases [6-8]. There is a local renin-angiotensin system (RAS) in the brain [9,10], and NADPH oxidase, AT1 and AT2 receptors have been located in dopaminergic (DA) neurons, nigral microglia and astrocytes [11-13].

We have previously shown that the DA cell loss induced by DA neurotoxins is enhanced by AII via AT1, activation of the microglial NADPH-complex and exacerbation of the glial inflammatory response [11,13,14]. This is consistent with more recent studies, in which we have shown hyperactivation of the nigral RAS in several animal models of increased vulnerability of DA neurons to degeneration (that is, models of humans at higher risk for PD), such as aged male rats [15] or menopausal rats [16]. The increased glial inflammatory response and DA neuron vulnerability were found to be inhibited by the AT1 antagonist candesartan. It is well-known that AT1 antagonists block AT1 receptor function and increase AT2 receptor expression and function with no significant changes in angiotensin converting enzyme (ACE) activity [17,18]. However, the mechanisms involved in the brain anti-inflammatory effects of AT1 blockers (ARBs) have not been clarified.

Previous studies in different tissues have suggested that peroxisome proliferator-activated receptor gamma (PPAR-γ) is involved in the anti-inflammatory effects of AT1 antagonists [19-21]. PPAR-γ belongs to a group of nuclear receptors (PPARs) that control lipid and glucose metabolism, energy homeostasis and adipocyte and macrophage differentiation. More recently, macrophage PPAR-γ receptors have been shown to be involved in the down-regulation of expression of several inflammatory cytokines and inhibition of inflammation [22-24]. Interestingly, PPAR-γ has been detected in neurons and glial cells [24-26], and participates in mechanisms that control microglial activation and lead to suppression of the activated phenotype [25,27]. In accordance, it has been shown that PPAR-γ agonists protect against DA cell death in animal models of PD [28,29]. However, the potential relationship between the anti-inflammatory effects of ARBs and PPAR-γ stimulation is not clear.

A number of studies have reported that some ARBs such as telmisartan and irbesartan, and more controversially losartan and candesartan (but not valsartan or olmesartan), have PPAR-γ activating properties that are independent of any AT1 blocking actions [19-21]. Therefore, the pharmacological PPAR-γ activating properties of ARBs may be responsible for the neuroprotective effects. However, it has also been reported that the pharmacological PPAR-γ-activating potency of ARBs (including telmisartan, the most potent PPAR-γ activator among ARBs) is rather modest compared with that of conventional PPAR-γ ligands, and that the PPAR-γ activating potency may be even less effective *in vivo *[30,31].

In the present study, we aimed to determine whether telmisartan provides neuroprotection against DA cell death in a mouse 1-methyl-4-phenyl-1,2,3,6-tetrahydropyridine (MPTP) model of parkinsonism, and whether PPAR-γ activation plays a major role in any such neuroprotection. Secondly, we studied whether the pharmacological PPAR-γ-activating properties of telmisartan are responsible for the neuroprotective effects, and if the AT1 blocking actions do not actually play any significant role in neuroprotection; we used AT1a-null mice lesioned with the DA neurotoxin MPTP to study whether deletion of AT1 in the absence of any pharmacological effect of ARBs provides neuroprotection. Thirdly, we investigated whether PPAR-γ activation may also play a major role in any such neuroprotective effect of AT1 deletion.

## Methods

### Experimental design

Male C57BL-6 mice weighing 20 to 25 g (that is, seven weeks old) were used. Mice were wild type (WT; Charles River, L'Arbresle, France) or homozygous mice deficient for AT1a (the major mouse AT1 isoform and the closest murine homolog to the single human AT1 [32]; Jackson Laboratory, Bar Harbor, ME, USA). Mice were maintained in the animal facility at the University of Santiago de Compostela in accordance with the institutional guidelines. In a first series of experiments, the WT mice were divided into seven groups (A1 to G1). Mice in group A1 (n = 14) were used as normal (that is, non-lesioned) controls, and were treated with vehicle (see below). Mice in group B1 (n = 11) were injected with MPTP (Free base, Sigma, St Louis, MO, USA; 30 mg/kg/day in saline, by intraperitoneal injection for five days) and intraperitoneal and oral vehicle. Mice in group C1 (n = 6) were injected with MPTP as group-B1 mice, but received oral treatment with telmisartan (5 mg/kg/day; Sigma) from two weeks before MPTP treatment until they were killed. The powered drug was administered orally to the mice mixed with peanut butter; animals in control groups were given only peanut butter. The dose of telmisartan was chosen on the basis of previous results. Telmisartan has been detected in cerebral spinal fluid after repeated oral treatment at 1 to 30 mg/kg [33]. However, the dose was selected according to several recent reports showing that 5 mg/kg provided neuroprotection against brain injury [34,35]. Mice in group D1 (n = 8) were injected with MPTP and telmisartan as above, as well as the PPAR-γ antagonist GW9662 (4 mg/kg by intraperitoneal injection in dimethyl sulfoxide 4% PBS for four weeks; that is from two weeks before MPTP injection until killed). Additional control mice were injected with telmisartan alone (group E1; n = 5), or GW9662 alone (group F1; n = 5), or telmisartan + GW9662 (group G1; n = 5) as described above.

In a second series of experiments, the AT1a-null mice were divided into four groups (A2 to D2). AT1a-null mice in group A2 (n = 8) were treated with vehicle and used as normal non-lesioned controls. Mice in group B2 and C2 (n = 8) were injected with MPTP as above. AT1a-null mice in group D2 (n = 8) were injected with MPTP and the PPAR-γ antagonist GW9662 (4 mg/kg by intraperitoneal injection for four weeks before killed). Finally, an additional group of AT1a-null mice was treated with GW9662 alone (group E2; n = 5). The mice were killed one week after treatment with MPTP or vehicle and then processed for histology or high performance liquid chromatography (HPLC; see below).

In a third series of experiments (n = 20), different groups of mice were injected with a single dose of MPTP (30 mg/kg) after treatment with vehicle or telmisartan as above (that is, WT mice + vehicle + MPTP, n = 7; WT mice + telmisartan + MPTP, n = 7; and AT1a-null mice + vehicle + MPTP, n = 6), and finally killed 90 min after the MPTP injection to quantify striatal levels of MPP^+ ^(see below) [36,37].

### High performance liquid chromatography

Seven days after the last MPTP injection, mice were killed by decapitation and brains rapidly removed. The striata were dissected on an ice-cold plaque, and the striatal tissue frozen on dry ice and stored at -80°C until analysis. Striatal tissue was homogenized and then centrifuged at 14,000 g for 20 min at 4°C. The supernatant fractions were decanted, filtered (0.22 μm) and injected (20 μL/injection) into the HPLC system (Shimadzu LC prominence, Shimadzu Corporation, Kyoto, Japan). Dopamine and its metabolites 3,4-dihydroxyphenylacetic acid (DOPAC) and homovanillic acid (HVA) were separated with a reverse phase analytical column (Waters Symmetry300 C18; 150 × 3.9 mm, 5 μm particle size; Waters, Milford, MA, USA). The mobile phase (70 mM KH_2_PO_4_, 1 mM octanesulfonic acid, 1 mM ethylenediaminetetraacetic acid (EDTA) and 10% MeOH, pH 4) was delivered at a rate of 1 mL/min. Detection was performed with a coulometric electrochemical detector (ESA Coulochem III, Chelmsford, MA, USA). The first and second electrode of the analytical cell were set at +50 mV and +350 mV, respectively; the guard cell was set at -100 mV. Data were acquired and processed with the Shimadzu liquid chromatography solution software. Results were expressed in nanogram per microgram wet weight tissue and presented as mean ± standard error of the mean (SEM) (n = 5 per group).

### Estimation of 1-methyl-4-phenylpyridinium levels by mass spectrometry

Brains were removed from the mice, the striata dissected on an ice-cold plaque and the striatal tissue frozen on dry ice and stored at -80°C until analysis. On the day of the assay [36], striata were weighed and sonicated in a solution of 0.4 M perchloric acid containing (w/v): 0.1% sodium metabisulphite, 0.01% EDTA and 0.1% L-cysteine. Samples were centrifuged at 13,000 rpm for 20 min at 4°C and the supernatant was used to determine 1-methyl-4-phenylpyridinium (MPP^+^) levels. HPLC separation was accomplished in a Waters Alliance 2795 system (Waters, Milford, MA, USA), with an Atlantis dC18 column (2.1 × 50 mm, 3 μm). The mobile phase consisted of solvent A (0.1% formic acid) and solvent B (acetonitrile). We employed an elution profile from 95% solvent A for 1 min, followed by a linear gradient from 95% solvent A to 100% solvent B from minute 1 to minute 1.5, and 100% solvent B was maintained until minute 5. A re-equilibration time of 5 min was allowed between injections and chromatography was carried out at a flow-rate of 0.2 mL/min. Eluates were detected with a Quattro MicroTM API ESCI triple-quadrupole mass spectrometer fitted with Z-spray (Waters, Milford, MA, USA). Electrospray ionization was set in positive ion polarizing mode (ESI+) for acquisition of mass spectrometry data, with the following fragments (m/z): 170.2 > 128.0, 170.2 > 154.4, and 170.2 > 115.1. The capillary voltage was set at 3 kV, the desolvation temperature at 450°C, the cone voltage at 45 V, and the desolvation gas flow rate was set at 550 L/h. All parameters were adjusted to obtain optimum operating conditions for maximum intensity of the selected fragments, with Masslynx 4.1 software (Waters, Milford, MA, USA). MPP^+ ^standards were prepared in the homogenization solution and used for calibration purposes.

### Immunohistochemistry, lectin histochemistry and cresyl violet staining of mouse brains

The animals were killed and perfused, firstly with 0.9% saline, and then with cold 4% paraformaldehyde in 0.1 M phosphate buffer, pH 7.4. The brains were removed, washed and cryoprotected in the same buffer containing 20% sucrose, and finally cut on a freezing microtome (30 μm thick). To prevent any possible unspecific labeling due to the use of primary mouse monoclonal antibodies with mouse tissue, sections were processed with rabbit polyclonal antibodies to tyrosine hydroxylase (TH; as a marker of DA terminals) and rat monoclonal antibodies against CD45 (to identify reactive microglia/macrophages), as follows. Sections were incubated for 1 h in 10% normal serum with 0.25% Triton X-100 in 20 mM potassium PBS containing 1% BSA (KPBS-BSA), then incubated overnight at 4°C with rabbit polyclonal antiserum to TH (Chemicon, Millipore Temecula, CA; 1:500) or at 4°C with rat monoclonal antiserum to CD45 (rat immunoglobulin G, 1:1000, AbD Serotec, Kidlington, Oxford, UK) in 20 mM KPBS containing 1% BSA, 2% normal serum and 0.25% Triton X-100. The sections were subsequently incubated, firstly for 90 min with the corresponding biotinylated secondary antibodies (1:200), and then for 90 min with an avidin-biotin-peroxidase complex (Vector, 1:50, Burlingame, CA, USA). Finally, the labeling was visualized with 0.04% hydrogen peroxide and 0.05% 3-3' diaminobenzidine (Sigma), containing 0.1% nickel sulfate to intensify the microglial staining. For negative control staining, sections were incubated in media lacking primary antibodies.

Activated microglial cells were also stained histochemically with *Griffonia simplicifolia *isolectin B4 (GSI-B4) as follows. Sections were pre-incubated in PBS containing 0.1 mM of CaCl_2_, MgCl_2_, MnCl_2 _and 0.3% Triton X-100 for 20 min. The sections were then rinsed with PBS and incubated overnight at 4°C with biotinylated GSI-B4 (Sigma; 20 μg/mL) in PBS containing cations and 0.3% Triton X-100. After rinsing with PBS, the sections were incubated with an avidin-biotin-peroxidase complex (Vector; 1:100) for 90 min. Finally, labeling was visualized with 0.04% hydrogen peroxide and 0.05% diaminobenzidine with 0.1% nickel sulfate to intensify the staining. For negative control staining, sections were incubated in media lacking GSI-B4.

The total numbers of TH-immunoreactivity (TH-ir) neurons in the substantia nigra compacta (SNc) were estimated by an unbiased stereology method (that is, the optical fractionator). Stereological analysis was carried out with the Olympus CAST-Grid system (Computer Assisted Stereological Toolbox; Olympus, Ballerup, Denmark). Uniform, randomly chosen sections through the substantia nigra (every third section) were analyzed for the total number of TH-ir cells by means of a stereological grid (fractionator), and the nigral volume was estimated according to Cavalieri's method [38]. Penetration by the antibody was determined by registration of the depth of each counted cell that appeared in focus within the counting frame. This analysis revealed incomplete penetration by the antibody, leaving 8 to10 μm in the center poorly stained [39]. The total number of cells was therefore calculated by excluding the volume corresponding to this portion of the sections.

In order to confirm that MPTP induces cell death and not only phenotypic down-regulation of TH activity, series of sections through the entire substantia nigra of control mice and mice treated with MPTP were counterstained with cresyl violet, and the total number of neurons in the SNc was estimated by the unbiased stereology method described above for TH-ir cells. Neurons were distinguished from glial cells on morphological grounds, and neurons with visible nuclei were counted as above. The number of reactive microglial cells was estimated with the Olympus CAST-Grid system and the unbiased stereological method described above for counting TH-ir neurons. The density of reactive microglial cells (cells/mm^3^) was determined by dividing the number of labeled cells by the volume that they occupied.

The density of striatal DA terminals was estimated as the optical density of the striatal TH-ir with the aid of National Institutes of Health (NIH)-Image 1.55 image analysis software (Wayne Rasband, National Institute of Mental Health, USA) on a personal computer coupled to a video camera (CCD-72, Imaging Research Inc, Linton, UK) and a constant illumination light table (Northern Light, St. Catharines, Canada). At least four sections through the central striatum of each animal were measured (both the right and left striatum), and for each section the optical densities were corrected by subtraction of background as observed in the corpus callosum.

### Statistical analysis

All data were obtained from at least three independent experiments and were expressed as means ± SEM. Multiple comparisons were analyzed by one-way analysis of variance (ANOVA) followed by the Student-Newman-Keuls post-hoc test. The normality of populations and homogeneity of variances were tested before each ANOVA. Differences were considered statistically significant at *P *< 0.05. Statistical analyses were carried out with SigmaStat 3.0 from Jandel Scientific (San Rafael, CA, USA).

## Results

**Figure 1:**
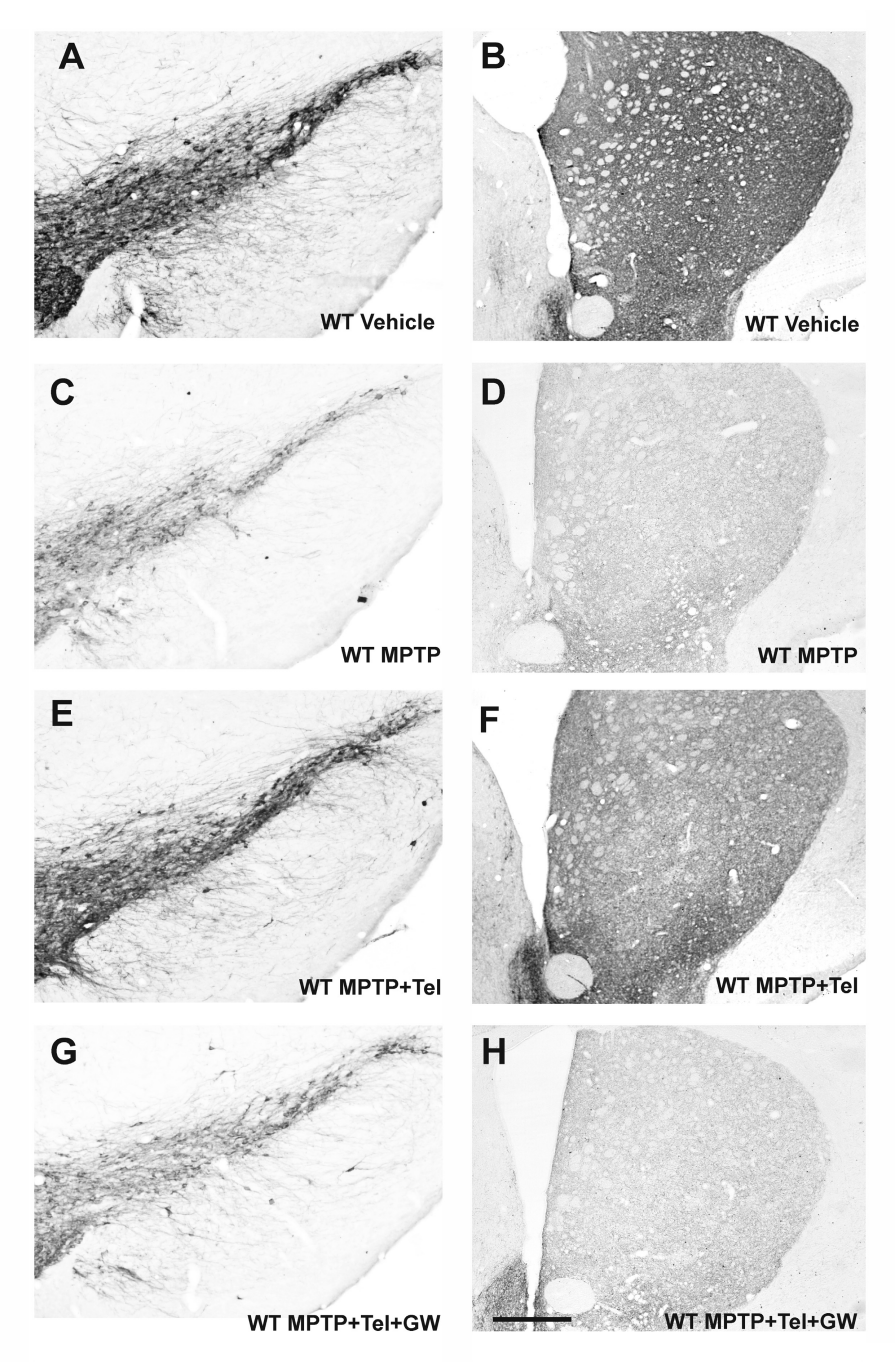
**Changes in TH-ir in the substantia nigra and striatum in WT mice**. TH-ir at central levels of the substantia nigra (**A**, **C**, **E**, **G**) and striatum (**B**, **D **,**F**, **H**) in WT mice injected with vehicle (controls; A, B), with MPTP alone (C, D), or with MPTP + telmisartan (E, F), or with MPTP + telmisartan + the PPAR-γ antagonist GW9662 (G, H). More TH-ir neurons were observed in the nigra and terminals in the striatum (that is, spared DA neurons and terminals) of mice treated with telmisartan (E, F) than in mice that did not receive telmisartan (C, D) or mice treated with telmisartan and GW9662 (G, H). Scale bar: 250 μm (A, C, E, G) and 560 μm (B, D, F, H).

**Figure 2:**
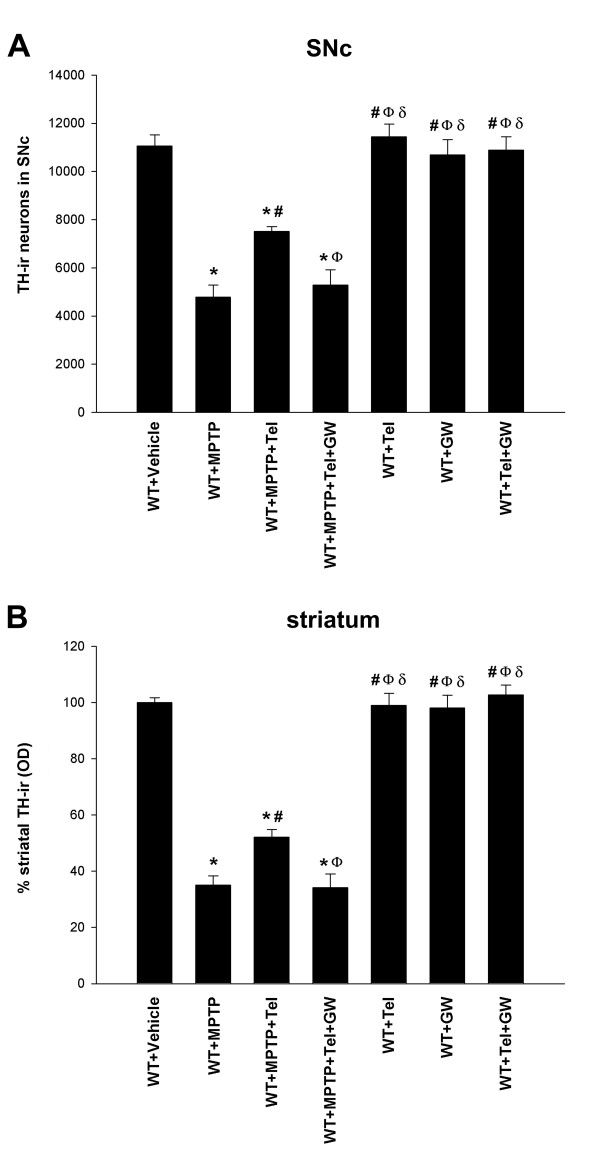
**DA (TH-ir) neurons and terminals in WT mice**. TH-ir neurons in the **(A) **SNc and **(B) **TH-ir terminals in the striatum one week after treatment with vehicle, telmisartan alone, GW9662 alone, telmisartan + GW9662, MPTP alone, telmisartan + MPTP, or telmisartan + MPTP + GW9662. The DA neurons were quantified as the total number of TH-ir neurons in the SNc, and density of striatal DA terminals was estimated as optical density and expressed as a percentage of the value obtained in the group treated with vehicle. Data are presented as mean ± SEM. *P < 0.05 compared with mice treated with vehicle, ^#^*P *< 0.05 compared with mice treated with MPTP alone, ^Ф^*P *< 0.05 compared with mice treated with MPTP + telmisartan, ^δ^*P *< 0.05 compared with mice treated with MPTP + telmisartan + GW9662 (one-way ANOVA and Student-Newman-Keuls post-hoc test).

In control mice (those not injected with MPTP; group A1) the DA neurons in the SNc were intensely immunoreactive to TH, and a dense evenly distributed TH-ir was observed throughout the striatum, indicating the presence of a dense network of nigrostriatal DA terminals (Figure 1A, B). In mice treated with MPTP and vehicle (group B1) there was a bilateral reduction in the number of TH-ir neurons in the substantia nigra and a marked reduction in the TH-ir in both striata relative to control mice (Figures 1C, D and 2A, B). The functional effects of the MPTP lesion were confirmed by determination of the striatal levels of dopamine and its metabolites with HPLC in control mice (group A1, n = 5) and mice treated with MPTP (group B1, n = 5). Levels (nanogram per milligram wet weight tissue) of dopamine (3.447 ± 0.243), DOPAC (0.257 ± 0.012) and HVA (0.336 ± 0.041) in control mice were significantly higher than those observed in lesioned mice (dopamine, 1.418 ± 0.112; DOPAC, 0.136 ± 0.012; HVA, 0.192 ± 0.024).

In order to confirm that MPTP induced DA cell death and not TH-down-regulation and the corresponding decrease in DA levels, we counted neurons in cresyl violet stained sections. In control mice, the total number of neurons counted in cresyl violet stained sections (13,701 ± 1140) was slightly higher than that of TH-ir neurons as some non-DA neurons located in the SNc were also counted. However, sections from mice treated with MPTP (group B1) showed significant fewer cresyl violet stained neurons in the SNc (8370 ± 1112) than in the control mice, confirming that MPTP induced cell death and not TH-down-regulation in the present experimental conditions.

Mice treated with telmisartan and injected intraperitoneally with MPTP (group C1) showed a bilateral reduction in the number of TH-ir neurons in the substantia nigra and density of striatal TH-ir terminals, relative to control mice, although the reduction was significantly lower than that observed in group B1 mice not treated with telmisartan (Figures 1E, F and 2A, B). However, the protective effects of telmisartan were inhibited by co-administration of the PPAR-γ antagonist GW9662 (group D1; Figures 1G, H and 2A, B). No significant changes were observed in mice treated with telmisartan alone, or GW9662 alone, or telmisartan + GW9662.

**Figure 3:**
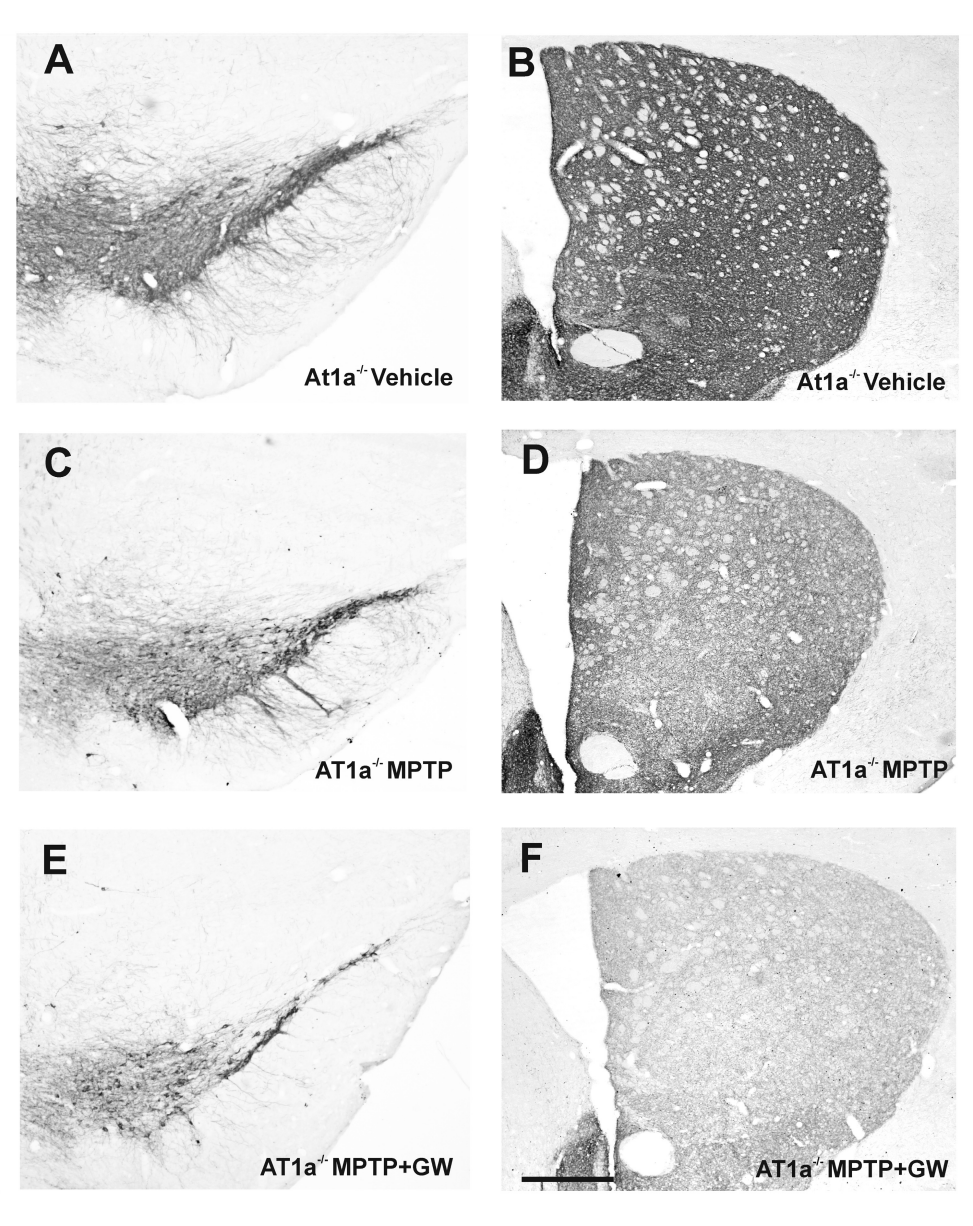
**Changes in TH-ir in the substantia nigra and striatum in AT1a-null mice**. TH-ir at central levels of the **(A, C, E) **substantia nigra and **(B, D, F) **striatum in AT1a-null mice (AT1a^-/-^) injected with vehicle (controls; A, B), with MPTP alone (C, D), or with MPTP + the PPAR-γ antagonist GW9662 (E, F). The number of TH-ir cells in the nigra and TH-ir terminals in the striatum (that is, spared dopaminergic neurons and terminals) was higher in the untreated group (C, D) than in mice treated with GW9662 (E, F). Scale bar: 250 μm (A, C, E) and 560 μm (B, D, F).

**Figure 4:**
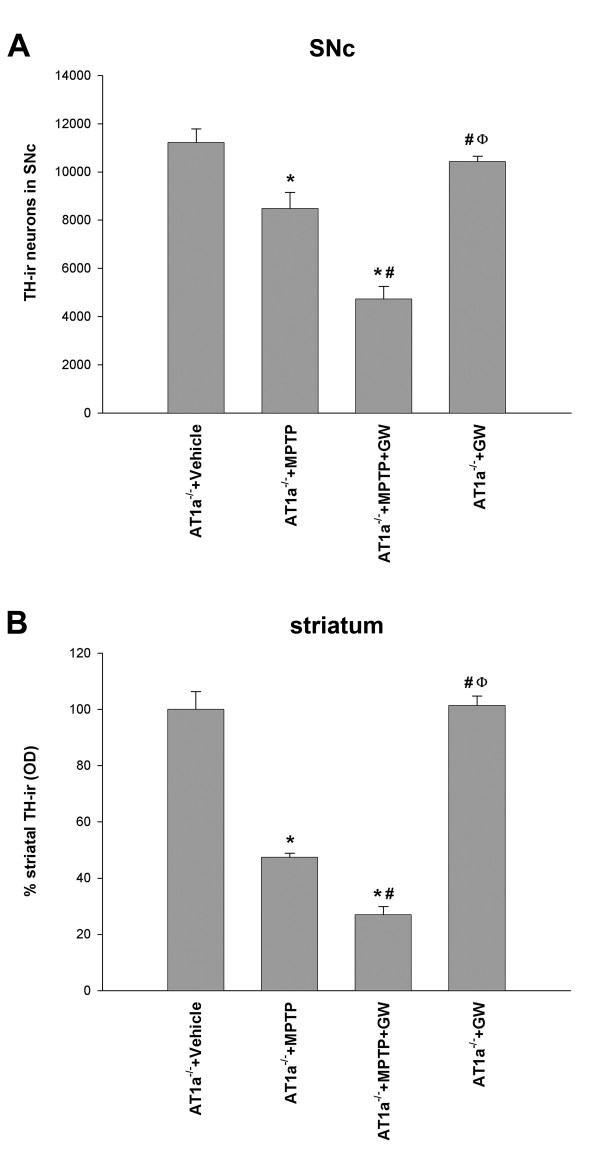
**DA (TH-ir) neurons and terminals in AT1a-null mice**. TH-ir neurons in the **(A) **SNc and **(B) **TH-ir terminals in the striatum one week after treatment with vehicle, GW9662 alone, MPTP alone, or MPTP + GW9662 in AT1a-null mice (AT1a^-/-^). The DA neurons were quantified as the total number of TH-ir neurons in the SNc, and density of striatal DA terminals was estimated as optical density and expressed as a percentage of the value obtained in the group treated with vehicle. Data are presented as mean ± SEM. *P < 0.05 compared with mice treated with vehicle, ^#^*P *< 0.05 compared with AT1a-null mice treated with MPTP alone, ^Ф^*P *< 0.05 compared with mice treated with MPTP + GW9662 (one-way ANOVA and Student-Newman-Keuls post-hoc test).

In control AT1a-null mice (those not injected with MPTP; group A2) DA neurons in the SNc were intensely immunoreactive to TH and a dense evenly distributed TH-ir was observed throughout the striatum (Figure 3A, B). In AT1a-null mice injected with MPTP (group B/C2) there was a bilateral reduction in the number of TH-ir neurons in the substantia nigra and their striatal terminals relative to vehicle-injected mice (Figures 3C, D and 4), although this reduction was lower than that observed in group B1 mice injected with MPTP and not subjected to AT1a deletion (that is, mice in which brain endogenous AII can act synergistically with MPTP on the DA system via AT1). However, the protective effects of AT1 deletion were inhibited by co-administration of the PPAR-γ antagonist GW9662 (group-D2 mice; Figures 3E, F and 4). No significant changes were observed in AT1a-null mice treated with GW9662 alone in comparison with mice treated with vehicle.

In order to determine if treatment with telmisartan or AT1a deletion acts by modifying MPTP pharmacokinetics such as penetration into the brain, biotransformation of MPTP to MPP^+ ^or MPP^+ ^removal from the brain, we measured striatal levels of MPP^+ ^in mice. There were no significant differences in striatal levels of MPP^+ ^between mice treated with telmisartan and MPTP (3.116 ± 0.196 ng/mg wet weight striatal tissue), AT1-null mice treated with vehicle and MPTP (3.100 ± 0.211 ng/mg wet weight striatal tissue) and WT mice treated with vehicle and MPTP (3.045 ± 0.157 ng/mg wet weight striatal tissue). The protective effect of telmisartan and AT1a deletion (that is, the absence of possible changes in the MPTP biotransformation to the active metabolite MPP^+^) was also supported by the results observed after treatment of mice with the PPAR-γ antagonist GW9662. In the presence of telmisartan or AT1 deletion (MPTP + telmisartan or MPTP + AT1 deletion), treatment with the PPAR-γ antagonist GW9662 reverted DA cell death and microglial activation (see below) to levels similar to those observed after treatment with MPTP alone, which would have not been possible without the presence of similar levels of MPP^+ ^in the mice striatum.

**Figure 5:**
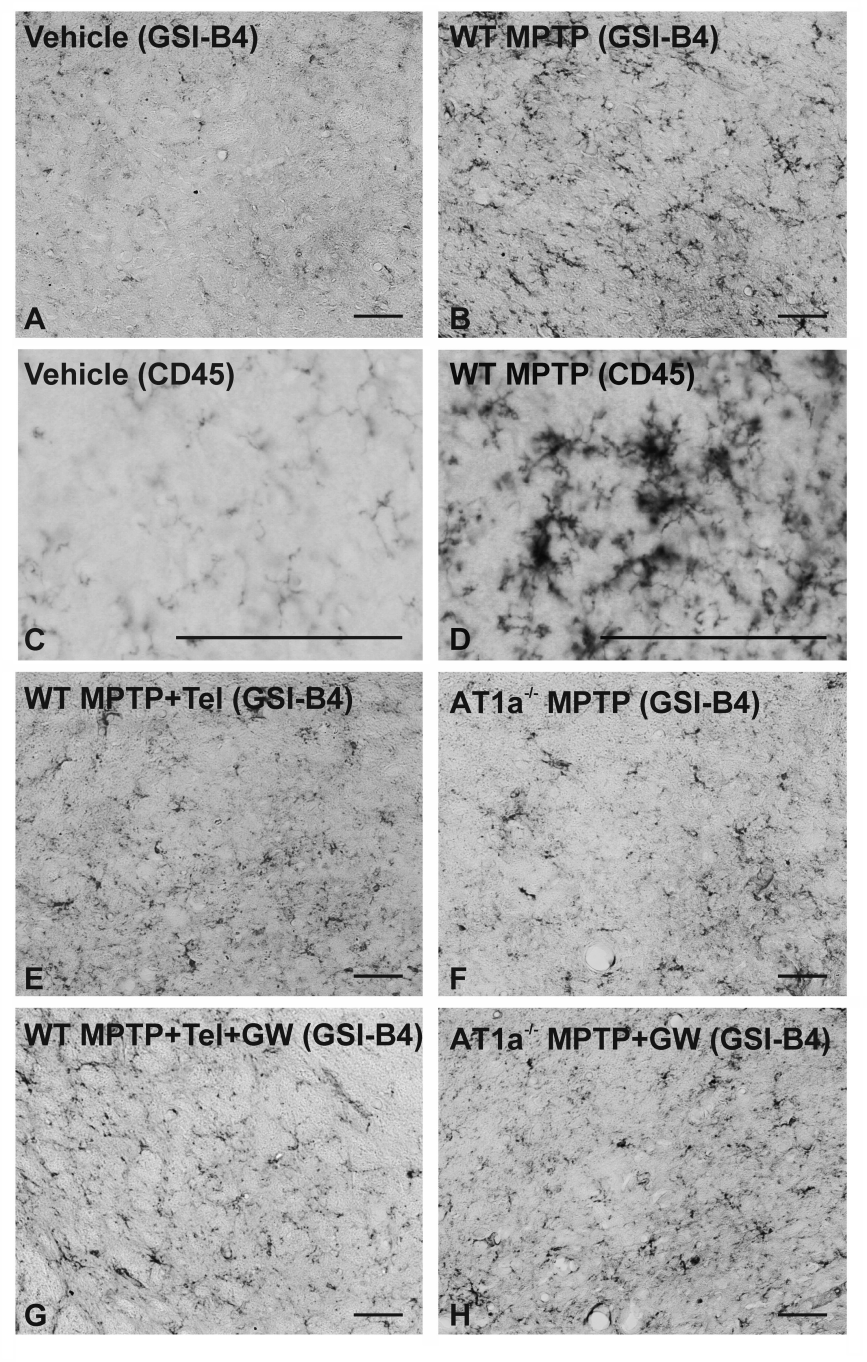
**Photomicrographs showing changes in microglial activation in the substantia nigra**. Activated microglial cells at central levels of the substantia nigra stained with **(A, B, E-H) **isolectin B4 or **(C, D) **immunostained for CD45 and higher magnification. Photomicrographs show microglia in WT mice treated with vehicle (controls; A, C), with MPTP alone (B, D), with MPTP + telmisartan (E), or with MPTP + telmisartan + GW9662 (G). Microglial activation in AT1a-null mice (AT1a^-/-^) treated with MPTP alone or MPTP + GW9662 is shown in F and H, respectively. Microglial activation was significantly higher in mice treated with MPTP alone (B, D), and in mice treated with the neurotoxin, AT1 inhibition and GW9662 (G, H). Scale bar: 100 μm.

**Figure 6:**
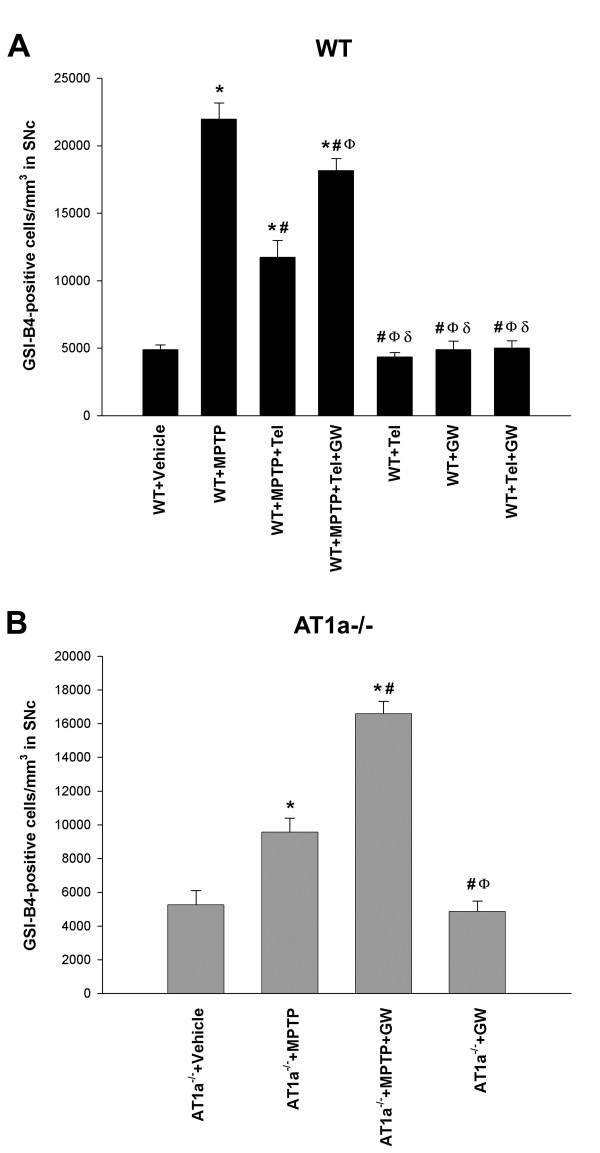
**Activated microglial cells in the SNc**. Density of GSI-B4-positive cells one week after treatment with **(A) **vehicle, telmisartan alone, GW9662 alone, telmisartan + GW9662, MPTP alone, MPTP + telmisartan, or MPTP + telmisartan + GW9662 in WT mice, and **(B) **vehicle, GW9662 alone, MPTP alone, or MPTP + GW9662 in AT1a-null mice (AT1a^-/-^). The microglial cells were quantified as the number of cells per mm^3^, and the data are presented as mean ± SEM. *P < 0.05 compared with WT mice (A) or AT1a-null mice (B) treated with vehicle, ^#^*P *< 0.05 compared with WT mice (A) or AT1a-null mice (B) treated with MPTP alone, ^Ф^*P *< 0.05 compared with WT mice treated with MPTP + telmisartan (A) or AT1a-null mice (B) treated with MPTP + GW9662, ^δ^*P *< 0.05 compared with WT mice treated with MPTP + telmisartan + GW9662 (one-way ANOVA and Student-Newman-Keuls post-hoc test).

In several recent studies, we have observed that the enhancing effect of AII on DA cell loss is mediated by microglial activation and exacerbation of the inflammatory response (for details, see [11,13]). In order to confirm that, in the present experiments, neuroprotection by telmisartan or AT1a deletion in mice is also associated with the same mechanism (inhibition of MPTP-induced microglial response), we analyzed the expression of the microglial markers isolectin B4 and CD45 in the substantia nigra. Control mice treated with vehicle showed minimal and non-significant microglial activation. In WT mice injected with MPTP (group B1), microglial activation was much higher than in WT mice injected with vehicle (group A1), and higher than mice injected with MPTP + telmisartan (group C1). However, WT mice injected with MPTP + telmisartan showed lower microglial activation than WT mice injected with MPTP + telmisartan + GW9662. No significant difference was observed between mice treated with vehicle and mice treated with telmisartan alone, or GW9662 alone, or telmisartan + GW9662 (Figures 5 and 6A).

In AT1-null mice injected with MPTP (group B/C2), microglial activation was higher than in AT1-null mice injected with vehicle, but significantly lower than in AT1-null mice treated with MPTP and the PPAR-γ antagonist GW9662. No significant difference was observed between AT1-null mice treated with vehicle and AT1-null mice treated with GW9662 alone (Figures 5F, H and 6B).

## Discussion

The present results show that, in mice, oral treatment with the ARB telmisartan protects nigral DA neurons against the DA neurotoxin MPTP as previously reported for other ARBs, such as candesartan and losartan [11,12]. This suggests that brain endogenous AII increases the neurotoxic effect of MPTP on the DA system, as observed in several previous studies, and that the AT1 blocker telmisartan inhibits the enhancing effect of AII on DA cell death. However, the protective effects of telmisartan were inhibited by co-administration of the PPAR-γ antagonist GW9662, which suggests that PPAR-γ activation is necessary for the neuroprotective effects of telmisartan to occur. This neuroprotective effect may be expected since telmisartan has been shown to be a potent AT1 blocker and to penetrate the blood-brain barrier to inhibit centrally mediated effects of AII [33,40]. However, the mechanism responsible for this neuroprotection has not been clarified. A first possibility is that the pharmacological PPAR-γ activating properties of ARBs are the only mechanism involved in the neuroprotective effect. Several studies have shown PPAR-γ activating properties of candesartan and losartan, and that among ARBs, telmisartan is the most potent agonist of PPAR-γ [19-21]. The present results are consistent with a major role of PPAR-γ activation as the data show that the protective effect of telmisartan was inhibited by co-administration of the PPAR-γ antagonist GW9662.

However, the present study shows that pharmacological PPAR-γ activating properties of ARBs are not the only factor responsible for neuroprotection; the results obtained with mice deficient in AT1 show that, independently of any pharmacological effect of ARBs, AT1 inhibition induces significant neuroprotection of DA neurons against neurotoxins such as MPTP. In fact, the neuroprotective effect of telmisartan against MPTP did not appear higher than that previously observed with candesartan [11], which has a less potent AT1-independent PPAR-γ agonistic effect [19-21]; this also suggests that there is no significant 'additional effect' of AT1 blockage and pharmacological PPAR-γ activating properties of ARBs. It is possible that the present experimental design was not able to reveal any possible additional effect. However, it may be also related to the PPAR-γ activating effect of the AT1 deletion observed in the present study; we observed that administration of GW9662 significantly increased the MPTP-induced DA neuron death in AT1 deficient mice, which suggests that PPAR-γ activation plays a major role in the neuroprotective effects of AT1 inhibition.

The results therefore suggest that inhibition of AT1 with ARBs, and with telmisartan in particular, leads to activation of PPAR-γ by a double mechanism that involves a pharmacological AT1-independent PPAR-γ agonistic effect (with more or less activation potency depending on the type of ARB) and a direct effect of the blockage of the AT1 itself, which also induces PPAR-γ activation. An important degree of crosstalk between RAS and PPAR-γ has been suggested in several studies carried out in different tissues [41,42]. It has been observed that treatment with AII inhibited PPAR-γ expression and the anti-inflammatory defense mechanisms in the artery wall [43,44]. In addition, inhibition of ACE led to enhanced expression of PPAR-γ in adipose tissue and skeletal muscle cells [45,46]. It has been suggested that AII inhibits PPAR-γ activation via AT1 and enhances PPAR-γ activation via AT2 receptors [42,47], and that AT2 receptors may gain functional importance during selective AT1 blockage by a redirection of the available AII to the AT2 receptor [47,48]. Conversely, a number of studies have suggested that PPAR-γ may modulate RAS and AII signaling at multiple levels [43]. PPAR-γ activators have been observed to induce down-regulation of AT1 expression [49-51] and ACE activity [52], and up-regulation of AT2 receptors [53].

Furthermore, other studies have shown that PPAR-γ and other PPARs may inhibit NADPH oxidase activity and other signaling pathways involved in AII-induced oxidative stress and inflammation [54,55]. This may explain not only the complete inhibition of the neuroprotective effect of telmisartan by the PPAR-γ antagonist GW9662, observed in the present study, but also the GW9662-induced inhibition of the neuroprotective effect of AT1 deletion in the AT1a-null mice. It is known that AII, via the AT2 receptor, exerts actions directly opposed to those mediated by AT1, thus antagonizing many of the effects of the latter [56,57]. In AT1a-null mice, AII may act via AT2 receptors activating PPAR-γ and contribute to inhibition of inflammation and oxidative stress, which has been observed to promote longevity and inhibit progression of degenerative diseases in AT1-null mice [58-60]. The present results, which showed that the protective effects of AT1 inhibition were blocked by the treatment with the PPAR-γ antagonist GW9662, are consistent with the latter findings.

In the present study, we have also confirmed that the mechanism involved in the observed neuroprotection is similar to that observed in previous studies on neuroprotective properties of ARBs. In previous studies in animal models of PD, we have shown that inhibition of microglial activation plays a major role in the protective effects of ARBs against DA cell death induced by DA neurotoxins [11,13,15]. The present results, which suggest that both AT1 inhibition with telmisartan and AT1a deletion inhibit the microglial response induced by MPTP in the substantia nigra, are consistent with this. Furthermore, the present results show a major role for the PPAR-γ activity in this effect, since treatment with the PPAR-γ antagonist GW9662 led to inhibition of the protective effect of telmisartan or AT1 deletion, as well as exacerbation of the microglial response induced by MPTP in the presence of AT1 inhibition. The present results are consistent with previous findings that showed that PPAR-γ activation down-regulates brain inflammation by inhibiting several functions associated with microglial activation [25,61], and that PPAR-γ agonists such as pioglitazone and rosiglitazone protect against MPTP-induced DA cell death by inhibition of microglial activation [28,29,62].

The present results are also consistent with studies that have observed that ARBs decreased the infiltration of CNS [63,64] and peripheral organs [65] by inflammatory cells, although some conflicting results have been also reported [66]. In accordance with their inhibitory effect on brain inflammation, beneficial effects of PPAR-γ agonists or AT1 inhibition have also been observed in a number of processes mediated by microglial activation and neuroinflammation, including animal models of Alzheimer's disease [67-69], brain ischemia [40,70,71], multiple sclerosis [63,64,72], traumatic brain injury [73] and aging [15,59,74].

In several previous studies we have shown the presence of AT1, AT2 receptors and NADPH oxidase in microglia and also in DA neurons [11,13]. In accordance with these findings, inhibition of neuronal AT1 receptors may decrease NADPH oxidase activity and NADPH oxidase-derived ROS in neurons, which may lead to direct inhibition of DA neuron death, followed by a subsequent reduction in microglial activation. However, our data do not suggest this possibility. In microglia and other inflammatory cells, NADPH oxidase produces ROS with dual functions. Firstly, high concentrations of ROS are released extracellularly to kill invading microorganisms or cells [75]. Secondly, low levels of intracellular ROS act as a second messenger in several signaling pathways involved in the inflammatory response [76,77]. In non-inflammatory cells, such as neurons, activation of NADPH oxidase stimulates production of low levels of intracellular ROS, which act as a second messenger in several signaling pathways, including those involved in triggering the inflammatory response and the migration of inflammatory cells into the lesioned area; NADPH oxidase-derived ROS may also modulate neuronal levels of ROS by interaction with mitochondrial derived ROS, and with ROS from other sources, such as neurotoxins or activated microglia. Cross-talk signaling between the NADPH oxidase and mitochondria has been observed in several types of cells. This includes not only an upstream role of NADPH oxidase in modulating of mitochondrial superoxide [78,79] but also that mitochondrial superoxide stimulates extramitochondrial NADPH oxidase activity in a feed-forward fashion [80,81]. This interaction was recently confirmed in a DA cell line treated with MPP^+ ^and angiotensin [82]; MPP^+ ^induced mitochondrial release of ROS, which induced a second wave of NADPH oxidase-derived ROS, which was reduced by treatment with the AT1 antagonist candesartan [82]. Using primary cultures of mesencephalic cells, we have previously shown that mitochondrial ATP-sensitive potassium channels play a major role in the interaction between NADPH-derived ROS and mitochondria after treatment with AII and/or DA neurotoxins such as MPP^+ ^or 6-hydroxydopamine [83,84].

However, we have also observed that only high doses of neurotoxins can induce DA neuron death in neuron-enriched primary mesencephalic cultures [11,13,83-85]. This was confirmed in a recent study using a DA cell line [82], in which significant DA cell death was only observed after treatment with very high doses of MPP^+ ^(300 μM). Interestingly, we observed that the effect of very low or sub-lethal doses of neurotoxins (0.25 μM MPP^+ ^or 10 μM 6-hydroxydopamine; in other words, more similar to the possible effect of environmental neurotoxins or other factors involved in PD) was enhanced by AII and induced significant DA cell death in mixed neuron-glia cultures but not in pure neuronal cultures (that is, in the absence of microglia) [11,13,83-85]. This suggests that although AII and ARBs may contribute to the modulation of intraneuronal ROS and neuronal release of pro-inflammatory signals, the microglial response plays a major role in the DA neuron death induced by low doses of neurotoxins, or other deleterious factors. The major role of ARBs inhibition of microglial reaction in reducing DA neuron death (rather than an ARBs-induced reduction in DA cell death resulting in a decreased microglial response) was also confirmed *in vivo *by the observation of an intense microglial response soon after a single injection of MPTP or 6-hydroxydopamine (that is, prior to significant DA neuron death), which was inhibited by treatment with ARBs [11,13,86,87]. The present study shows that ARBs-induced PPAR-γ activation plays a major role in this effect.

## Conclusion

The results of the present study show that oral administration of telmisartan produces effective neuroprotection against DA cell death induced by MPTP, as previously observed for candesartan, and that the neuroprotective effect is mediated by PPAR-γ activation. Furthermore, the results in AT1-deficient mice show that the deletion of AT1, which is unrelated to the pharmacological properties of ARBs, protects against the DA neurotoxin, and that the protective effects of AT1 deletion are also inhibited by PPAR-γ blockage. The results suggest that inhibition of AT1 with ARBs, and with telmisartan in particular, leads to activation of PPAR-γ by a double mechanism that involves a pharmacological AT1-independent PPAR-γ agonistic effect (with more or less activation potency depending on the type of ARB) and a direct effect of the blockage of the AT1 itself, which also induces PPAR-γ activation.

## Abbreviations

ANOVA: analysis of variance; ACE: angiotensin-converting enzyme; AII: angiotensin II; ARBs: AT1 blockers; AT1: angiotensin type 1 receptor; AT1a^-/-^: AT1a-null mice; AT2: angiotensin type 2 receptor; BSA: bovine serum albumin; DA: dopaminergic; DOPAC: 3,4-dihydroxyphenylacetic acid; EDTA: ethylenediaminetetraacetic acid; GSI-B4: *Griffonia simplicifolia *isolectin B4; HPLC: high performance liquid chromatography; HVA: homovanillic acid; KPBS-BSA: potassium PBS containing 1% BSA; MPP^+^: 1-methyl-4-phenylpyridinium; MPTP: 1-methyl-4-phenyl-1,2,3,6-tetrahydropyridine; NADPH: nicotinamide adenine dinucleotide phosphate; PBS: phosphate-buffered saline; PD: Parkinson's disease; PPAR γ: peroxisome proliferator-activated receptor gamma; RAS: renin-angiotensin system; ROS: reactive oxygen species; SEM: standard error of the mean; SNc: substantia nigra compacta; TH: tyrosine hydroxylase; TH-ir: TH-immunoreactivity; WT: wild-type.

## Competing interests

The authors declare that they have no competing interests.

## Authors' contributions

PG-G conducted the experiments, participated in the statistical analysis and drafted the manuscript. BJ performed the histological study. AIR-P performed the stereological analysis and participated in the statistical analysis. MJG and JLL-G conceived the study and its design, supervised the project and edited the manuscript preparation. All authors read and approved the final manuscript.
